# T-Cell Receptor β Chain and B-Cell Receptor Repertoires in Chronic Hepatitis B Patients with Coexisting HBsAg and Anti-HBs

**DOI:** 10.3390/pathogens11070727

**Published:** 2022-06-26

**Authors:** Qiao Zhan, Le Chang, Jian Wu, Zhiyuan Zhang, Jinghang Xu, Yanyan Yu, Zhenru Feng, Zheng Zeng

**Affiliations:** 1 Department of Infectious Diseases, Peking University First Hospital, Beijing 100034, China; dralettazhan@bjmu.edu.cn (Q.Z.); ddcatjh@sina.com (J.X.); 2 Department of Clinical Laboratory, Peking University First Hospital, Beijing 100034, China; changleyeah@163.com; 3 MyGenostics Inc., Beijing 101318, China; jw2231@mygeno.cn (J.W.); zhangzhiyuan@mygeno.cn (Z.Z.)

**Keywords:** high-throughput sequencing, immune repertoire, chronic hepatitis B, HBsAg, anti-HBs, coexistence

## Abstract

Antibodies in response to antigens are related to the immune repertoire of T- and B-cell receptors. However, some patients with chronic hepatitis B (CHB) have coexisting HBsAg and anti-HBsAg antibodies (anti-HBs) that cannot neutralize HBV. We attempted to investigate the repertoires that produce this response in CHB patients. The T-cell receptor β chain (TRB) and B-cell receptor (BCR) repertoires of peripheral blood genomic DNA were analyzed using MiXCR. T-cell receptor (TCR) cluster analysis was carried out by clusTCR, and motifs prediction was selected by Multiple Em for Motif Elicitation (MEME). A total of 76 subjects were enrolled, including 26 HBsAg and anti-HBs coexisting patients with CHB (DP group), 25 anti-HBs single-positive healthy people (SP group), and 25 CHB patients (CHB group). The clone length of BCR in 39, 90 was significantly different among these groups (*p* = 0.005, 0.036). The motif “CASSLG” in the DP group was significantly higher than SP and CHB groups and may relate to coexistence, and the motif “GAGPLT” was only shown in the SP group and may relate to anti-HB expression. These provide important insights into vaccine development and CHB treatment.

## 1. Introduction

Chronic hepatitis B (CHB), caused by the hepatitis B virus (HBV), is one of the most common chronic infectious diseases worldwide, affecting 296 million individuals in 2019 [1]. There were 709,419 individuals that died from HBV-related liver diseases globally in 2017, such as liver cirrhosis and hepatocellular carcinoma; Asian regions accounted for 75.3% of these global disability-adjusted life-years (DALYs) [2]. In the serological detection and diagnosis of CHB, hepatitis B surface antigen (HBsAg) is the most important marker, which indicates current HBV infection and can be used as a follow-up marker. Hepatitis B surface antibody (anti-HBs) is a specific protective antibody that can neutralize HBsAg. Positive anti-HBs indicate HBV infection recovery. However, cases of simultaneous HBsAg and anti-HBs have been frequently reported and discovered during clinical practice that is difficult to explain by the typical serological patterns.

Arnold et al. first reported the coexistence of HBsAg and anti-HBs with different subtypes both in serologic and fluorescence histologic studies in 1976 [3]. Even after more than 40 years of research, the mechanisms underlying this serological pattern have not been well delineated and understood. Jiang et al. recently reviewed three aspects of the overall possible mechanisms, which include mutations in the viral genome, immune status and genetic factors of the hosts, and impact of methodology [4]. With the immune pressures coming from antiviral treatment and vaccination, HBV mutations are not limited to open reading frames (ORFs) but are also found in all viral genes and regulatory elements clustered in the preS/S gene, reverse transcriptase region (RT) in the polymerase gene, the pre-core region, basal core promoter (BCP), and the X gene, resulting in the reduced or even abolished binding of neutralizing antibodies and eliciting the detection of HBsAg and anti-HBs at the same time [5,6,7,8,9,10,11,12,13,14,15]. In terms of host factors, the presence of heterologous subtype-specific anti-HBs along with human gene oligoadenylate synthetase 3 (OAS3) variants may be associated with the coexistence of HBsAg and anti-HBs [16,17,18,19].

Existing mechanism studies on the coexistence of HBsAg and anti-HBs in HBV infection mainly focus on viruses, ignoring the host’s immune response as well as the amounts and categories of expressed antibodies. Immune repertoire analysis is a novel detection technique that uses the entire range of human T-cell receptors (TCRs) and B-cell receptors (BCRs) of the adaptive immune system to reveal the immune response statement. According to the TCR genes, there are two types of T cells, αβT cells, and γδT cells, that are, respectively, composed of alpha chains (TRA), beta chains (TRB) and gamma chains (TRG), and delta chains (TRD). The variable region of each chain consists of three complementarity-determining regions (CDRs), which are variable and determine the antigen specificity. Among the three CDRs, CDR3 is generated by random selection and recombination of variable (V), diversity (D), and joining (J) genes [20,21]. With the development of more and more immune repertoire analyses in HBV infection [22,23], high-throughput sequencing of the immune repertoire may be helpful in understanding the immune statement and mechanism in HBV infection.

Therefore, we conducted a systematic analysis of TRB and BCR immune repertoires in peripheral blood samples in CHB patients with coexisting HBsAg and anti-HBs to identify the immune mechanisms related to this uncommon phenomenon and further clarify a novel path toward a complete HBV cure.

## 2. Results

### 2.1. Patient Characteristics

In this study, we recruited 76 subjects and categorized them into the DP group (CHB patients with HBsAg and anti-HBs coexisting) (*n* = 26), SP group (HBsAg negative and anti-HBs positive healthy people or hepatitis B cured) (*n* = 25), and CHB group (HBsAg positive and anti-HBs negative CHB patients) (*n* = 25). The characteristics of the patients are shown in Table 1. No significant differences in age or gender were found among the three groups. In the DP group, 6 of 26 patients were HBV-DNA negative, and the others had a 4.51 log10 IU/mL HBV-DNA load on average. The HBeAg positive ratio was 73.08%. Of the 26 patients, 20 were HBV-DNA positive and were sequenced in the HBV P region, and 6 patients were found with 7 HBV gene mutations, including 145R, 130N, 126S, 145R, 119R, T116A, and I126S. In the SP group, the median anti-HBs value was 125.02 IU/L. In the CHB group, 9 of 25 patients were HBV-DNA negative and had a 4.12 log10 IU/mL HBV-DNA load on average. Only two patients had sequenced HBV with no mutations. The HBeAg positive ratio was 40%. Compared to DP and CHB groups, we found that the HBsAg in the DP group was significantly higher than that in the CHB group (*p* = 0.001) mainly because the detection up-limit of HBsAg in nine of the CHB group patients was 250 IU/mL and these HBsAg data were counted as 250 IU/mL for statistical analysis.

### 2.2. Immune Repertoire Profiling

**Figure 1:**
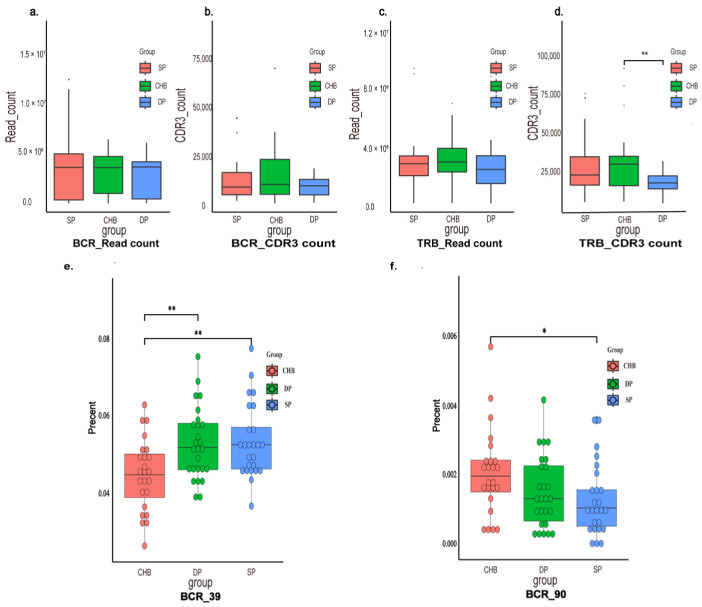
Sequence profiles of the immune repertoires. The upper histograms show the read counts (**a**,**c**) and CDR3 counts (**b**,**d**) of BCR and TRB. The CDR3 counts of TCR in DP group were lower than SP group (**d**). The lower histograms show the CDR3 length distribution of immune repertoires in three groups. The frequency of CDR3 sequence in BCR with 39 nucleotides increased significantly in DP group compared with CHB group (**e**) and 90 nucleotides decreased in SP group compared with CHB group (**f**). *p*-values < 0.05, or <0.01 were denoted by “*”, and “**”, respectively. Abbreviations: CDR3, complementarity-determining region 3; BCR, B-cell receptor; TRB, T-cell receptor β chain; DP, double positive; SP, single positive; CHB, chronic hepatitis B.

A total of 460,630,687 clean reads were obtained from 76 subjects. Each TRB had an average of 2,800,911 clean reads, 46,375 clones, and 26,442 CDR3 counts, and each BCR had an average of 3,260,019 clean reads, 30,784 clones, and 26,442 CDR3 counts per sample. The sequence profiles of the immune repertoires are shown in Supplementary Table S1. Figure 1 shows that the CDR3 counts of TRB in the DP group were lower than in the CHB group (*p* = 0.004). The frequency of CDR3 sequences with unique length nucleotides showed differences among the three groups (Supplementary Table S2). The frequency of the CDR3 sequence in BCR with 39 nucleotides increased significantly in the DP group compared with the CHB group (*p* = 0.005), and 90 nucleotides decreased in the SP group (*p* = 0.036) (Figure 1e,f). However, the distribution patterns of the CDR3 lengths in TRB were not different among the three groups. Taken together, the above data suggested that there is an increase in TRB clonotypes, along with the clonotypes of CDR3 in BCR, with clonal expansion of lengths of certain kinds of nucleotides in the DP group.

### 2.3. Diversity Analysis

**Figure 2:**
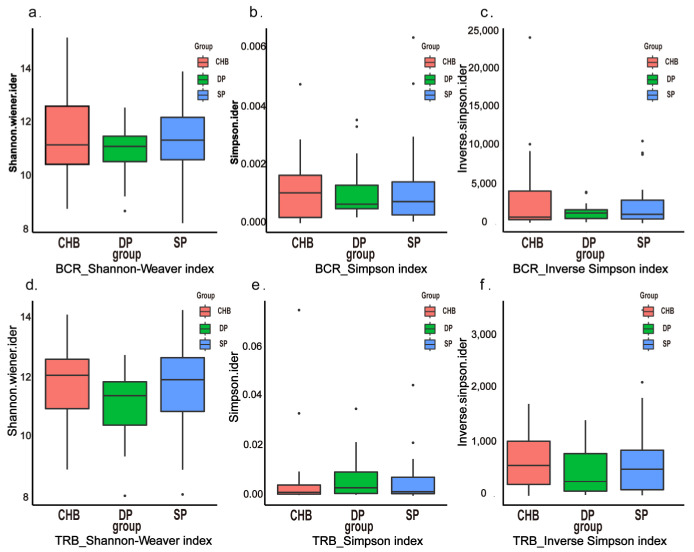
Diversity analysis of immune repertoires. Longitudinal analysis of Shannon index (**a**,**d**), Simpson index (**b**,**e**), and inverse Simpson index (**c**,**f**) for BCR and TRB in the DP, SP, and CHB groups. Abbreviations: BCR, B-cell receptor; TRB, T-cell receptor β chain; DP, double positive; SP, single positive; CHB, chronic hepatitis B.

Based on the clean data and immune repertoire profile described above in the three groups, the characteristics of the diversity of BCR and TRB were analyzed using common diversity indexes, including the Shannon index, Simpson index, and inverse Simpson index. No significant differences were noted in the Shannon index, Simpson index, and inverse Simpson index of TRB and BCR (Figure 2a–f).

### 2.4. Analysis of V, D, and J Gene Segments

**Figure 3:**
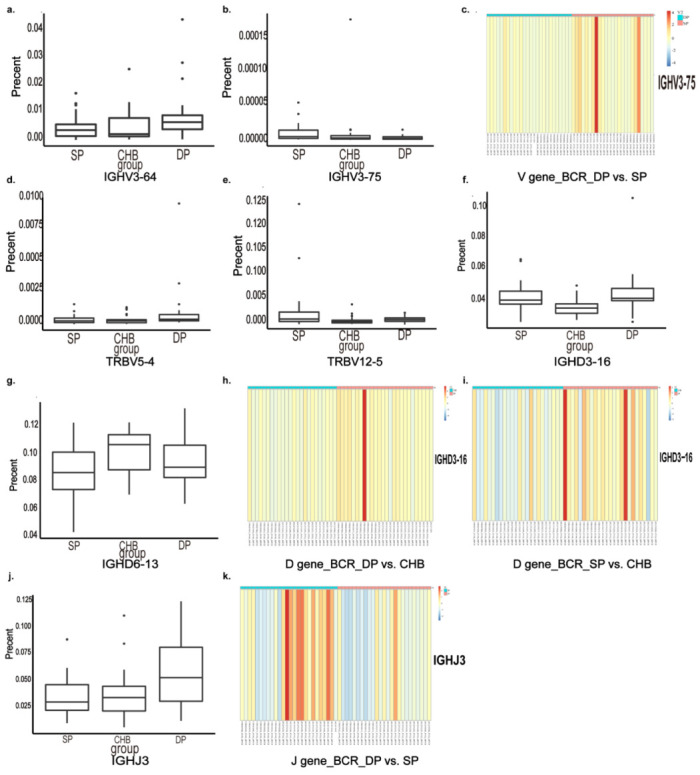
Analysis and comparison of differential genes of V, D, and J gene segments. V genes were significantly differentially expressed among three groups shown in the histogram (**a**,**b**,**d**,**e**) and heatmap (**c**) (DP group in color blue and SP group in color red). IGHV3-64, IGHV3-75 of BCR (**a**–**c**), TRBV5-4, TRBV12-5 of TRB (**d**,**e**) were recognized. D genes were significantly differentially expressed among three groups shown in the histogram (**f**,**g**) and heatmaps (**h**,**i**). CHB group in color blue and DP group in color red in Figure 3h, and CHB group in color blue and SP group in color red in Figure 3i. IGHD3-16 and IGHD6-13 of BCR were recognized. J genes were significantly differentially expressed among three groups shown in the histogram (**j**) and heatmap (**k**) (DP group in color blue and SP group in color red). IGHJ3 was recognized. The use frequency of V, D, J gene fragments is shown by the heatmap bar (log2(FC) > 1, *p* < 0.017). Abbreviations: BCR, B-cell receptor; TRB, T-cell receptor β chain; DP, double positive; SP, single positive; CHB, chronic hepatitis B.

The comparison of V, D, and J gene fragments in TRB and BCR among the three groups is shown in Figure 3. Regarding V gene expression, the frequency of IGHV3-64 and IGHV3-75 of BCR was significantly different among the three groups as compared in the histograms (Figure 3a,b) (*p* = 0.034, *p* = 0.026, respectively). The heatmap shows that the expression of gene IGHV3-75 was low in the DP group compared with that in the SP group (*p* = 0.009) (Figure 3c). Analysis revealed differentially expressed V genes of TCR, including TRBV5-4 and TRBV12-5, among the three groups (Figure 3d,e) (*p* = 0.030, *p* = 0.049, respectively).

The D gene segment is located between the V and J regions, intercepted from the end of the V zone to the beginning of the J zone. We demonstrated the differential D gene expression in TRB and BCR and found the expression of genes IGHD3-16 and IGHD6-13 were significantly different among the three groups (*p* = 0.0003, *p* = 0.04, respectively) (Figure 3f,g). IGHD3-16 was more highly expressed in the DP group than the CHB group in pair-wise comparisons in the three groups (*p* = 0.0004) (Figure 3h). This gene was also more highly expressed in the SP group than in the CHB group, as shown in the heatmap (*p* = 0.001) (Figure 3i). No differentially expressed D gene was found in TRB among the three groups.

Regarding J gene expression, the frequency of IGHJ3 of BCR was significantly increased in the DP group compared with the SP and CHB groups (*p* = 0.010), as shown in Figure 3j. Nevertheless, there were no significant differential gene fragments in TRB among the three groups.

### 2.5. Analysis of V–J, V–D–J Gene Combinations, and Amino Acid Clonotypes

**Figure 4:**
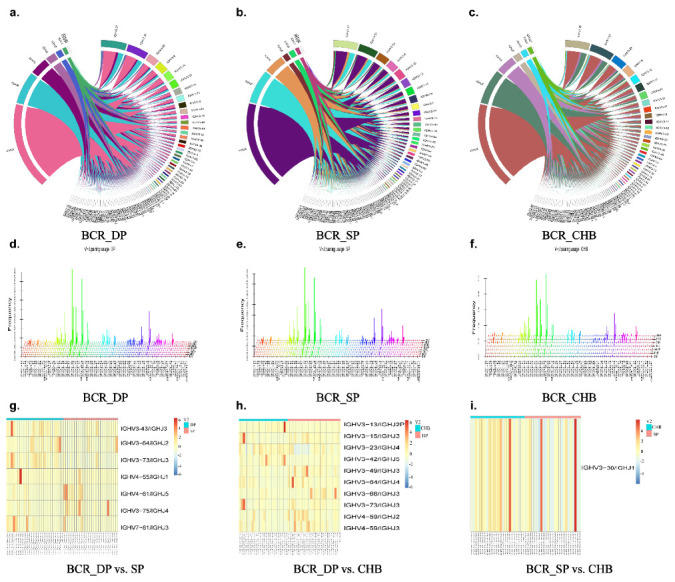
Analysis and comparison of differential genes of V–J gene combinations in BCR. Circos figures (**a**–**c**) showed the V–J combination visually. Each color block represents one gene, and the breadth of the block reflects the frequency. The wider the color block, the higher the frequency. Three-dimensional (3D) images (**d**–**f**): *x*-axis shows IGHV genes and *z*-axis shows IGHJ genes. Heatmap of significantly differential V–J combinations of BCR in DP group vs. SP group, DP group vs. CHB group, and SP group vs. CHB group (log2(FC) > 1, *p* < 0.017) (**g**–**i**). Abbreviations: BCR, B-cell receptor; DP: double positive; SP: single positive; CHB: chronic hepatitis B.

**Figure 5:**
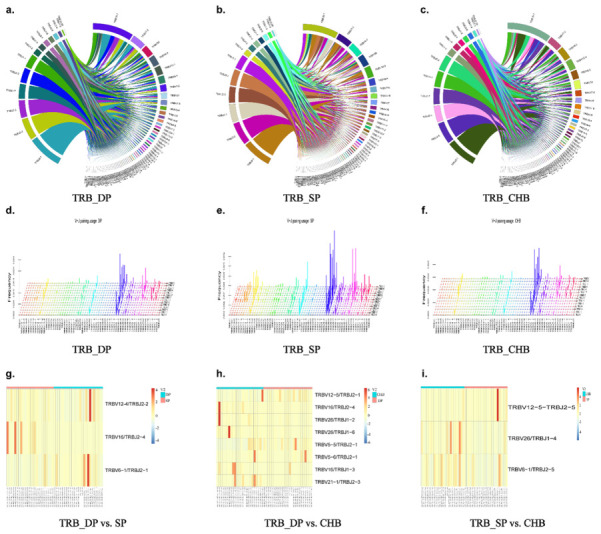
Analysis and comparison of differential genes of V–J gene combinations in TRB. Circos figures (**a**–**c**) showed the V–J combination visually. Each color block represents one gene, and the breadth of the block reflects the frequency. The wider the color block, the higher the frequency. Three-dimensional (3D) images (**d**–**f**): *x*-axis shows TRBV genes and *z*-axis shows TRBJ genes. Heatmap of significantly differential V–J combinations of TRB in DP group vs. SP group, DP group vs. CHB group, and SP group vs. CHB group (log2(FC) > 1, *p* < 0.017) (**g**–**i**). Abbreviations: TRB, T-cell receptor β chain; DP, double positive; SP, single positive; CHB, chronic hepatitis B.

We further analyzed and compared the expression of V–J paired genes among the three groups shown by the Circos figures, three-dimensional (3D) images, and heatmaps (Figure 4 and Figure 5). Circos figures and 3D images play an important role in the analysis of the V–J combination of immune repertoire sequencing, which can intuitively display the combination of V–J genes, the dominant position of some specific genes, as well as the extent and frequency of TRB and BCR diversity through visual images. In the Circos figures, each color block represents one gene, and the breadth of the block reflects the frequency. The wider the color block, the higher the frequency. Therefore, we produced Circos figures and 3D images of the TRB and BCR to observe the usage of BCR bearing a combination of IGHV with IGHJ genes, as well as the usage of TRB bearing a combination of TRBV with TRBJ genes, as shown in Figure 5. The frequency of combinations of V–J genes of TRB and BCR differed among the three groups. From BCR heatmaps, we found that the V–J gene combinations IGHV3-43/IGHJ3, IGHV3-64/IGHJ2, IGHV3-73/IGHJ3, IGHV4-55/IGHJ1, IGHV3-75/IGHJ4 were expressed higher in DP group than that in the SP group. Meanwhile, V–J gene combinations IGHV4-61/IGHJ5 and IGHV7-81/IGHJ3 were expressed higher in the SP group than DP group. Eight V–J gene combinations, IGHV3-13/IGHJ2P, IGHV3-15/IGHJ3, IGHV3-49/IGHJ3, IGHV3-64/IGHJ4, IGHV3-66/IGHJ3, IGHV3-73/IGHJ3, IGHV4-59/IGHJ2, and IGHV4-59/IGHJ3, were significantly higher expressed in the DP than CHB groups. Gene IGHV3-23/IGHJ4 and IGHV3-42/IGHJ5 were expressed higher in the CHB group than in the DP group. The expression of IGHV3-30/IGHJ1 was lower in the SP group than in the CHB group (*p* = 0.012). Heatmaps shown in Figure 5 demonstrate the different TRB V–J gene combinations expressed among the three groups compared in pairs. We found lower expressions of TRBV12-4/TRBJ2-2 and TRBV16/TRBJ2-4 in the DP group than in the SP group (*p* = 0.006, *p* = 0.004, respectively); the expression of TRBV6-1/TRBJ2-1 was higher in the DP group (*p* = 0.015). Five significantly high expressed V–J genes were found in the DP group than the CHB group, including TRBV12-5/TRBJ2-1, TRBV5-5/TRBJ2-1, TRBV5-6/TRBJ2-1, TRBV16/TRBJ1-3, and TRBV21-1/TRBJ2-3. V–J gene combinations TRBV16/TRBJ2-4, TRBV26/TRBJ1-2, and TRBV26/TRBJ1-6 were expressed higher in the CHB group than in the DP group (Figure 5h). Figure 5i showed that the gene TRBV12-5-TRBJ2-5 was expressed higher in the SP group than CHB group, while genes TRBV26-TRBJ1-4 and TRBV6-1-TRBJ2-5 were expressed higher in CHB group.

**Figure 6:**
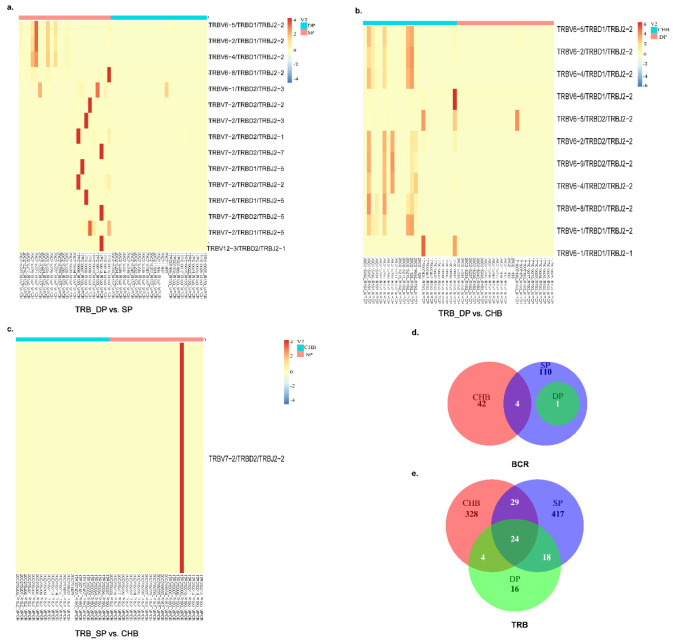
Analysis and comparison of differential genes of V–D–J gene combinations. Heatmap of significant differential V–D–J combinations of TRB (**a**–**c**) in DP group vs. SP group, DP group vs. CHB group, and SP group vs. CHB group (log2(FC) > 1, *p* < 0.017). Wayne diagrams showed meaningful differential CDR3 clonotypes among groups in the BCR and TRB analysis (**d**,**e**). Abbreviations: BCR, B-cell receptor; TRB, T-cell receptor β chain; DP, double positive; SP, single positive; CHB, chronic hepatitis B.

We next investigated the V–D–J combination to further identify differentially expressed genes as well as corresponding amino acid clonotypes and narrowed down the diversity of the immune repertoire among the three groups (Figure 6). For the BCR subset, no significantly differentially expressed V–D–J combinations were found in the three groups in pair-wise comparisons. As for TCR, the heatmaps in Figure 6a showed that there were 15 differentially expressed V–D–J combinations between the DP and SP groups. Eleven differentially expressed V–D–J combinations between the DP and CHB groups were demonstrated in Figure 6b. The specific *p*-values of differentially expressed genes and corresponding clone types are shown in Supplementary Table S3. Gene TRBV7-2/TRBD2/TRBJ2-2 was more highly expressed in the SP group than in the CHB group (*p* = 0.009). Wayne diagrams showed meaningful differential CDR3 clonotypes among groups (Figure 6d,e). Overall, there were significant differences regarding the V–D–J combinations and amino acid clonotypes of the immune repertoire among HBsAg and anti-HBs simultaneously positive CHB patients (DP group) and two controls.

### 2.6. Principal Component Analysis

To find the best factor for distinguishing HBsAg and anti-HBs simultaneously positive CHB patients from HBsAg-positive CHB patients and anti-HBs positive, healthy subjects, we compared V genes, Ds gene, J genes, V–D combinations, and V–D–J combinations among the three groups by principal component analysis (Supplementary Figure S1). There was no difference in TRB and BCR among the DP, SP, and CHB groups.

### 2.7. TCR Clusters Analysis and Motifs Prediction

**Figure 7:**
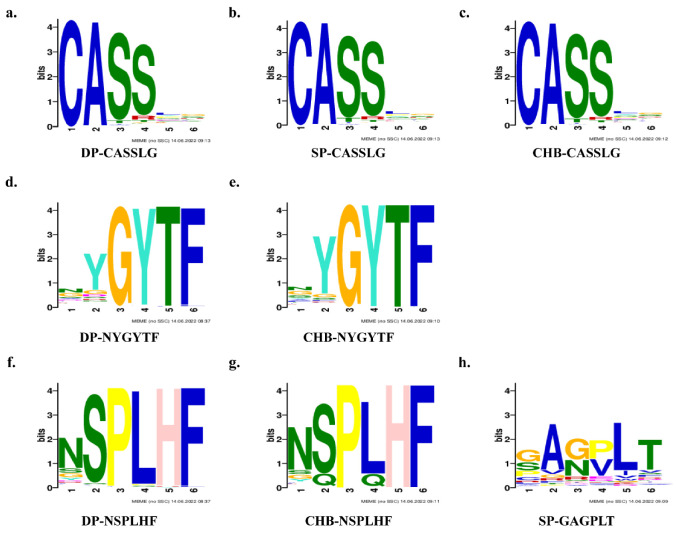
Motif prediction. Motif logos “CASSLG” of DP, SP, and CHB groups (**a**–**c**), motif “NYGYTF” and “NSPLHF” in DP and CHB groups (**d**–**g**) and motif “GAGPLT” in SP group (**h**) were analyzed by MEME. The label on the *x*-axis represents the positions of the CDR3 amino acid and *y*-axis represents the bits. Abbreviations: TRB, T-cell receptor β chain; DP, double positive; SP, single positive; CHB, chronic hepatitis B.

To identify the TRB motif and its potential function in HBV infection, we analyzed the TRB clusters and then predicted the motifs. The top 10 TRB clusters, as evaluated by clusTCR in the three groups, are shown in Supplementary Figure S2 [24]. Next, to elicit motifs among the three groups, we imported the whole CDR3 amino acid of TRB into an online tool MEME and obtained five predicted motifs for each group, including “CASSLG” in identical common (Table 2) [25]. Motif “CASSLG” was selected based on sequence conservation and E-value, and the logos in three groups are demonstrated in Figure 7a–c, which would relate to the HBV infection. We further analyzed the amount and proportion of CDR3 amino acid, which contained the motif “CASSLG” in three groups and found that there were 19.36% (647,282/392,326) in the DP group, 10.21% (444,611/500,000) in the SP group, and 11.74% (506,593/500,124) in the CHB group. The proportion of the CDR3 amino acid containing motif “CASSLG” in the DP group was significantly higher than SP and CHB groups, which indicated that the motif “CASSLG” may be concerned with HBsAg and anti-HBs coexistence in HBV infection. Furthermore, motif “NYGYTF” and “NSPLHF” was predicted both in the DP and CHB group, which would establish a bridge in the HBV-specific motif (Figure 7d–g). Moreover, the motif “GAGPLT” was shown separately in the SP group, indicating that this motif would play an important role in anti-HBs production after HBV vaccination and cure (Figure 7h).

## 3. Discussion

CHB caused by HBV is one of the most prevalent infectious diseases globally. Clinical recovery is considered when the patient is anti-HBs positive. However, some patients still have persistent HBV infection even when anti-HBs are positive. According to the large sample of epidemiological studies in different regions of the world, the prevalence rate of HBV infection with HBsAg and anti-HBs coexisting is about 2–10% [8,9,12,26,27,28]. This special phenomenon has important clinical significance. First of all, in the absence of simultaneous detection of HBsAg, anti-HBs, and other serological markers, the coexistence of HBsAg and anti-HBs may lead to missed diagnosis and misdiagnosis of HBV infection. Secondly, anti-HBs cannot be used to determine whether such patients have acquired hepatitis B-related immunity. Importantly, some studies have suggested that the coexistence of HBsAg and anti-HBs may be associated with cirrhosis and liver cancer. Heijtink et al. found that CHB patients with HBsAg and anti-HBs simultaneous positivity had a higher frequency of liver cancer compared with chronic hepatitis B patients with only HBsAg positivity [29]. Multivariate analysis showed that HBsAg and anti-HBS positivity was a risk factor for chronic HBV infection to develop liver cancer.

This mechanism of HBsAg and anti-HBs coexistence remains poorly understood. There are mainly three hypotheses, including mutations in the viral genome, immune state, and genetic factors of the hosts. Mutations in different ORFs of HBV, including mutations in the preS/S gene, especially the “a” determinant, the transcriptase region, and overlapping preC/BCP and X genes, can change the stability and immunogenicity of HBsAg, favoring the selection of immune escape variants that contribute to the coexistence of HBsAg and anti-HBs [8,10,30]. Antiviral treatments, vaccination, and even persistent HBV infection can lead to the accumulation of these variants [11,31,32,33]. The presence of heterologous subtype-specific anti-HBs, particularly in clinical immunosuppression, should be considered in the immune mechanism [16,17,18]. Host genetic factors also play a role, and the human gene oligoadenylate synthetase 3 (OAS3) variants may be associated with the coexistence of HBsAg and anti-HBs [19].

Considering the heterogeneity between studies, including different study populations, inclusion and exclusion criteria, sensitivity and specificity of commercial assay kits, HBV infection conditions, and genotypes, the results from different studies are not exactly consistent and comparable. The current HBsAg and anti-HBs double-positive studies mainly focus on the viral mutation but not the host immune response. A few studies on host response were focused on the host genetics but not the immune system reaction. To identify the host immune response involved in the coexistence of HBsAg and anti-HBs in CHB patients, we analyzed the immune repertoire, a novel immune research strategy to reflect the immune status of simultaneously positive HBsAg and anti-HBs CHB patients.

Our study used high-throughput sequencing to evaluate the immune repertoire, which included TRB and BCR in peripheral blood of HBsAg and anti-HBs simultaneously positive CHB patients, HBsAg-positive CHB patients, and anti-HBs-positive healthy subjects. We found that the number and length of the immune repertoires were different in HBsAg and anti-HBs simultaneously positive CHB patients, including the decreased CDR3 counts of TRB. Chen et al. demonstrated that mutations I92T, F93C, and C95W occurred in cytotoxic lymphocyte (CTL) epitopes of S proteins and led to higher amino acid variation compared to the HBsAg- alone group, impairing the recognition sites for immune cells, and preventing CTL activation, which partly explains the decreased CDR3 counts of TRB in the DP group [12]. Furthermore, clonotypes of CDR3 in BCR with certain kinds of nucleotide lengths had clonal expansion among patients with coexisting HBsAg and anti-HBs.

Our study also revealed the immune repertoire in terms of V genes, D genes, J genes, V–J combinations, V–D–J combinations, and amino acid clonotypes. We found some different expressions of V, D, and J genes, including IGHV3-64, IGHV3-75, TRBV5-4, TRBV12-5, TRAV14DV4, IGHD3-16, IGHD6-13, and IGHJ3, indicating that the corresponding cells, especially B lymphocytes that express these genes, expanded differently in patients with coexisting HBsAg and anti-HBs. Meanwhile, there were significant differences regarding the V–J combinations, V–D–J combinations, and amino acid clonotypes of the immune repertoire in CHB patients with coexisting HBsAg and anti-HBs and controls, providing a new direction for early recognition and therapeutic targets for CHB.

Furthermore, we utilized some sequence prediction tools to identify consensus sequences and motifs. We found a de novo motif “CASSLG” as a consensus sequence that would relate to anti-HBs antibody production to explain the HBsAg and anti-HBs coexistence phenomenon. Moreover, the motif “NYGYTF” and “NSPLHF” could be the HBV-specific motif, and the motif “GAGPLT” may relate to anti-HBs production, which paves the way for HBV vaccination and treatment. Yang et al. have reported that the motifs “NTE,” “QETQ,” and “GG-Q (E)-ETQ” were expressed mostly in BV27 and BV7-9, which are significantly increased in hepatitis B vaccine responders compared to those in non-responders in the TRB repertoire analysis of hepatitis B vaccine response [34]. Furthermore, the motif “KLNSPL” was found in nearly 80% of CDR3s in BV27/J1-6 from hepatitis B vaccine responders. Although the study population and analysis methods were different in our studies, antibody production is the common factor whether in response to HBV natural infection or hepatitis B vaccination. Maru et al. analyzed the TCRα and TCRβCDR3 in HBeAg positive CHB patients and found that the most frequent rearrangement Vß2s1-D1-Jß1.1 had two kinds of CDR3 amino acid motifs including “YICS TRTGD TEAFFGQGTRLVV” and “YICSAR DRGD TEAFFGQGTRLVV” [35]. These motifs would be related to early-stage HBV infection. Furthermore, the Vα7.2 gene segment of TCRα was most frequently used (16.4%), joining with the Jα33 gene, which demonstrated the role of αTCR in HBV infection. In our study, the αTCR repertoire of three groups needs to be further sequenced in the following research work. More verification experiments in vitro and in vivo are needed to further screen and validate these findings.

Our study has some limitations. We mainly focused on immune response without the human leukocyte antigens (HLA) analysis as well as the sorted adaptive immune cells repertoire, and more data are needed. HLA allotypes are distinctive from individual to individual, leading to different immune patterns in different individuals against HBV. The polymorphism of HLA Alleles is inextricably linked with HBV infection, which needs to be further analyzed in our future immune repertoire sequencing of sorted CD4+T cell, CD8+ T cell, γδT cell, and B cells.

In summary, we found that there were significant differences related to the TRB and BCR repertoire, especially the differentially expressed genes of BCR between CHB patients with coexisting HBsAg and anti-HBs and controls. The motif “CASSLG” and “GAGPLT” was also identified for further research. These findings may help to develop antiviral therapy and cure chronic HBV infection.

## 4. Materials and Methods

### 4.1. Patients

A total of 76 subjects aged 18–65 were recruited from Peking University First Hospital (Beijing, China), including 26 patients with coexisting HBsAg and anti-HBs with CHB (DP group), 25 anti-HBs single positive, healthy people (SP group), and 25 HBsAg positive CHB patients (CHB group). Patients with superinfection or co-infection with hepatitis A, C, D, or E virus, cytomegalovirus, human immunodeficiency virus, autoimmune disorders, immunosuppressive treatment, end-stage liver insufficiency, or malignancies were excluded. The βTCR and BCR repertoires were detected in all three groups. HBV markers, including HBsAg, anti-HBs, HBeAg, anti-HBe, and anti-HBc, were detected by electrochemiluminescence in Cobas e 601 (Roche, Basel, Switzerland) and followed up for at least 6 months. HBV-DNA was detected by PCR-fluorescence in COBAS TaqMan 48 (Roche, Basel, Switzerland). Antiviral therapy was received by 62% of the DP group and 50% of the CHB. This study was approved by the Ethical Committee of Peking University First Hospital and conformed to the provisions of the Declaration of Helsinki. All subjects provided written informed consent.

### 4.2. DNA Extraction and Immune Repertoire Library Construction

Peripheral blood samples were obtained from 76 subjects. DNA was extracted from whole blood using a QIAamp DNA Blood Mini Kit (NO. 51306, Qiagen, Hilgen, Germany), and the concentration was tested using NanoDrop 2000 spectrophotometer (Thermo Fisher Scientific, Waltham, MA, USA). The DNA was used as a template for PCR amplification, which was performed to generate the library of TCR and BCR. The step 1 PCR amplification protocol was as follows: 95 °C for 5 min; 95 °C for 30 s, 59 °C for 30 s; 72 °C for 1 min for 30 cycles; and 72 °C for 10 min. The step 2 PCR amplification protocol was as follows: 98 °C for 2 min; 98 °C for 30 s, 65 °C for 30 s; 72 °C for 30 s for 10 cycles; and 72 °C for 5 min. The PCR products were purified, and the barcodes were confirmed. The annealed region of these primers was the CDR3 region, as shown by Multiplex PCR Amplifies. The CDR3 regions were sequenced on the Illumina X platform (MyGenostics, Beijing, China). According to the sequencing depth, the test covered about 90% of the TCR or BCR. The CDR3 regions were identified based on the definition established by the International ImMunoGeneTics (IMGT) (http://www.imgt.org/, accessed on 19 March 2021) collaboration, and the V, D, and J segments contributing to each CDR3 region were identified by a standard algorithm [36].

### 4.3. Data Processing and Sequence Analysis

The original data were converted to raw sequence reads and low-quality sequences discarded, and then data were stored in FASTQ format. We used MiXCR software (v3.0.12, MiLaboratories Inc, Sunnyvale, CA, USA)to identify the V, D, and J genes, extracted the CDR3 sequence and corrected PCR errors to get clean reads [37]. The productive immune repertoire sequence reads were filtered by removing (a) any reads with CDR3s shorter than four amino acids; (b) CDR3 contigs with a length that was not a multiple of 3; (c) contigs containing stop codons. The diversity of TRB and BCR was analyzed by normalized Shannon diversity entropy (NSDE), Simpson index, and inverse Simpson index [38]. Equations of the diversity indices are as follows:

$$Shannon\;index=-\overset{n}{\underset{i-1}{\sum }}Pi\times \ln\left(Pi\right)\;$$

$$Simpson\;index=1-\overset{n}{\underset{i=1}{\sum }}Pi^{2}$$

$$Inverse\;Simpson\;index=\frac{1}{\lambda }=\overset{n}{\underset{i=1}{\sum }}Pi^{2}\;$$

Circular plots were created using Circos to reflect the recombination of V–J pairs [39].

### 4.4. Clustering Estimation and Motifs Prediction of TRB Clonotypes

In order to identify the same or similar antigen specificity clonal groupings, we analyzed the TRB repertoire of all patients using a reported clustering tool named clusTCR that is available as an anaconda package (https://anaconda.org/svalkiers/clustcr, accessed on 27 October 2021) [24]. The clusTCR works in two steps, one to allow fast and efficient clustering and a second to perform accurate clustering.

Following the clustering analysis by clusTCR, we next elicited the motifs by an online tool named MEME version 5.4.1 (https://meme-suite.org/meme/tools/meme, accessed on 20 December 2021) in order to search for novel signals in sets of TCR protein sequences in the three groups of patients [25]. MEME was designed based on an algorithm that discovers one or more motifs in a collection of DNA or protein sequences by using the technique of expectation maximization to fit a two-component finite mixture model to the sets of sequences, and it estimates how many times each motif occurs in each sequence in the dataset and outputs an alignment if the motif occurs [40]. We selected the classic motif discovery mode, input our primary sequences of TCR protein of three groups in FASTA format, and selected the site distribution zero or one occurrence per sequence to find three motifs per subject. The E-value, sites, width, and logo were all included in the HTML output of the results.

### 4.5. Statistical Analysis

R software (R Foundation for Statistical Computing, Vienna, Austria) was used to identify the construction and expression of the different genes. The normal distribution data were described as mean ± standard deviation, and the skewed distribution data were described as median (25 quantiles, 75 quantiles). The Mann–Whitney U test and Wilcoxon test were used for two-group comparisons, while the Kruskal–Wallis H test was used among three groups. Principal component analysis (PCA) was used for clustering analysis. All data were analyzed using two-tailed tests, and differences with *p*-values less than 0.05 in the three-group comparison as well as a *p*-value less than 0.017 in the three groups compared in pairs were considered statistically significant. *p*-values < 0.05, < 0.01, or <0.001 were denoted by “*”, “**”, and “***”, respectively.

## Figures and Tables

**Table 1:** Clinical characteristics of subjects.

Group	DP (26)	SP (25)	CHB (25)	*p*-Value
Gender, male, % (*n*)	57.69 (15)	52.00 (13)	60.00 (15)	0.841
Age, years	38.38 ± 11.82	40.28 ± 8.01	40.68 ± 8.51	0.278
HBeAg positive ratio, % (*n*)	73.08 (19)	-	40.00 (10)	0.017
HBeAg (COI)	14.69 (0.48–1150.25)	-	0.40 (0.22–302.88)	0.121
HBsAg (IU/mL)	2578.00 (1186.00–6989.75)	-	250.00 (250.00–1731.00)	0.001
Anti-HBs (IU/mL)	100.92 (35.11–249.18)	125.02 (25.05–401.83)	-	0.474
HBV DNA (log10 IU/mL)	4.51 ± 2.06	-	4.12 ± 2.14	0.466

Notes: There were 62% in the DP group and 50% in the CHB group who received antiviral therapy. The normal distribution data (age and HBV DNA) were described as mean ± standard deviation, and the skewed distribution data (HBeAg, HBsAg, and anti-HBs) were described as median (25 quantiles, 75 quantiles). Abbreviations: HBV, hepatitis B virus; HBeAg, hepatitis B e antigen; HBsAg, hepatitis B surface antigen; Anti-HBs, hepatitis B surface antibody; DP, double positive; SP, single positive; CHB, chronic hepatitis B; COI, cutoff index.

**Table 2:** Predicted motifs of TRB in three groups.

Group	Sequence	Width	Sites	E-Value
DP	SSGGGGNEQ	9	347,562	1.7 × 10^−1963^
	CASSLG	6	75,967	3.2 × 10^−206^
	NYGYTF	6	21,764	4.8 × 10^−063^
	NSPLHF	6	4350	8.6 × 10^−008^
	NTEAFF	6	3965	1.1 × 10^+001^
SP	SSGGGGNEQ	9	499,849	1.2 × 10^−1887^
	CASSLG	6	51,033	2.0 × 10^−083^
	ANYGYT	6	379	1.1 × 10^+006^
	YYGYTF	6	166	2.4 × 10^+006^
	GAGPLT	6	89	2.4 × 10^+006^
CHB	SSGGGGNE	8	485,120	1.5 × 10^−1783^
	CASSLG	6	58,698	1.3 × 10^−064^
	NSPLHF	6	4194	1.8 × 10^−024^
	SYEQYF	6	12,060	1.8 × 10^−017^
	NYGYTF	6	12,270	2.7 × 10^−013^

Notes: Different motifs were predicted by MEME using CDR3 amino acid sequences of three groups. The E-value of a motif is based on its log-likelihood ratio, width, sites, the background letter frequencies, and the size of the training set. The lower the E-value, the more significant the difference. Abbreviations: TRB, T cell receptor β chain; DP: double positive; SP: single positive; CHB: chronic hepatitis B.

## Data Availability

The datasets generated and/or analyzed during the current study are not publicly available as they are still being investigated but are available from the corresponding author (Zheng Zeng) upon reasonable request.

## Supplementary Materials

The following supporting information can be downloaded at: https://www.mdpi.com/article/10.3390/pathogens11070727s1, Figure S1: Sequence profiles of immune repertoires.; Figure S2: Cluster analysis. Sequence logos of annotated top 10 clusters βTCR clonotypes analyzed by clusTCR in DP (a), SP (b), and CHB (c) groups; Table S1: The frequency of CDR3 sequences with unique length nucleotides; Table S2: The frequency of CDR3 sequences with unique length nucleotides; Table S3: Different V–D–J combinations and CDR3 amino acid clonotypes of TRB. (*p* < 0.017)
