# Supplementary material for: T-Cell Receptor β Chain and B-Cell Receptor Repertoires in Chronic Hepatitis B Patients with Coexisting HBsAg and Anti-HBs
Source: Pathogens. 2022 Jun 26;11(7):727. doi: 10.3390/pathogens11070727 (PMC9318409; doi:10.3390/pathogens11070727)
Supplement: Supplementary file 1 [file pathogens-11-00727-s001.zip › pathogens-1751438-supplementary.pdf]

Supplementary Material

Supplementary Table S1. Sequence profiles of immune repertoires.

Immune repertoires	Group	Subjects	$\bar{x} \pm s$	total
TRB	CHB	Read_count	3112225.30±1813644.04	74693407
		Clone_count	56384±34099.32	1353216
		CDR3_count	33219.46±21591.38	797267
	SP	Read_count	3052632.75±2283806.38	73263186
		Clone_count	50831.38±33196.93	1219953
		CDR3_count	30337.46±20122.01	728099
	DP	Read_count	2596506.32±1779414.20	64912658
		Clone_count	35013.44±13401.03	875336
		CDR3_count	19368.56±6591.57	484214
BCR	CHB	Read_count	3269980.25±2201949.73	78479526
		Clone_count	36572.08±32616.96	877730
		CDR3_count	16204.17±15355.95	388900
	SP	Read_count	3468181.92±3361464.83	86704548
		Clone_count	33741.12±35027.80	843528
		CDR3_count	13767.56±12104.90	344189
	DP	Read_count	3176052.38±2922643.02	82577362
		Clone_count	23780.77±14287.45	618300
		CDR3_count	9941.77±4866.70	258486

Notes: The read count, clone count, and CDR3 count were demonstrated in $\bar{x} \pm s$ format of BCR and TRB in DP, SP, CHB groups. Abbreviations: CDR3, complementarity-determining region 3; BCR, B-cell receptor; TRB, T cell receptor β chain; DP: double positive; SP: single positive; CHB: chronic hepatitis B

Supplementary Table S2. The frequency of CDR3 sequences with unique length nucleotides.

immune repertoires	nucleotides length	DP	SP	CHB	Kruskal value	P value
BCR	39	0.052	0.053	0.045	10.50	0.01
BCR	90	0.001	0.001	0.002	6.63	0.04

Notes： The frequency and P values of CDR3 sequences with unique length nucleotides were demonstrated in Supplementary Table S2. Abbreviations: BCR, B-cell receptor; DP: double positive; SP: single positive; CHB: chronic hepatitis B

Supplementary Table S3. Different V-D-J combinations and CDR3 amino acid clonotypes of TRB. (P<0.017)

Group	V/D/J combination	CDR3 amino acid clonotypes	P value
DP vs. SP	TRBV6-2/TRBD1/TRBJ2-2	CASSYLGNTGELFF	0.0005
	TRBV6-5/TRBD1/TRBJ2-2	CASSYLGNTGELFF	0.0009
	TRBV6-4/TRBD1/TRBJ2-2	CASSYLGNTGELFF	0.0030
	TRBV6-8/TRBD1/TRBJ2-2	CASSYLGNTGELFF	0.0042
	TRBV6-1/TRBD2/TRBJ2-3	CASSEPRRLAGPTDTQYF	0.0110
	TRBV7-2/TRBD1/TRBJ2-5	CASSYAGGSTYEQYF	0.0087
	TRBV7-2/TRBD2/TRBJ2-2	CASSSGLAGGMENTGELFF	0.0088
	TRBV7-2/TRBD2/TRBJ2-3	CASSLSGSVSGTQYF	0.0147
	TRBV7-2/TRBD2/TRBJ2-1	CASSLVRRGEQFF	0.0147
	TRBV7-2/TRBD2/TRBJ2-7	CASSFGASGSYEQYF	0.0089
	TRBV7-2/TRBD2/TRBJ2-2	CASSPNFLGELFF	0.0089
	TRBV7-8/TRBD1/TRBJ2-5	CASSLERGDEQYF	0.0089
	TRBV7-2/TRBD2/TRBJ2-5	CASSPSRLAASYNEQYF	0.0089
	TRBV7-2/TRBD1/TRBJ2-5	CASSLDATEAYEQYF	0.0080
	TRBV12-3/TRBD2/TRBJ2-1	CASSLGGEQYF	0.0089
DP vs. CHB	TRBV6-2/TRBD1/TRBJ2-2	CASSYLGNTGELFF	0.0000
	TRBV6-5/TRBD1/TRBJ2-2	CASSYLGNTGELFF	0.0001
	TRBV6-4/TRBD1/TRBJ2-2	CASSYLGNTGELFF	0.0001
	TRBV6-6/TRBD1/TRBJ2-2	CASSYLGNTGELFF	0.0006
	TRBV6-8/TRBD1/TRBJ2-2	CASSYLGNTGELFF	0.0009
	TRBV6-2/TRBD2/TRBJ2-2	CASSYVGNTGELFF	0.0014
	TRBV6-1/TRBD1/TRBJ2-2	CASSYLGNTGELFF	0.0020

	TRBV6-5/TRBD2/TRBJ2-2	CASSYVGNTGELFF	0.0023
	TRBV6-9/TRBD2/TRBJ2-2	CASSYVGNTGELFF	0.0030
	TRBV6-4/TRBD2/TRBJ2-2	CASSYVGNTGELFF	0.0033
	TRBV6-1/TRBD1/TRBJ2-1	CASSPGQGVYNEQFF	0.0089
SP vs. CHB	TRBV7-2-TRBD2-TRBJ2-2	CASSWALAGGGELFF	0.0089

Notes：The differential expression V-D-J combinations and corresponding CDR3 amino acid clonotypes of TRB with P value < 0.017 between each two groups were shown in Supplementary Table S3. Abbreviations: CDR3, complementarity-determining region 3; TRB, T cell receptor β chain; DP: double positive; SP: single positive; CHB: chronic hepatitis B

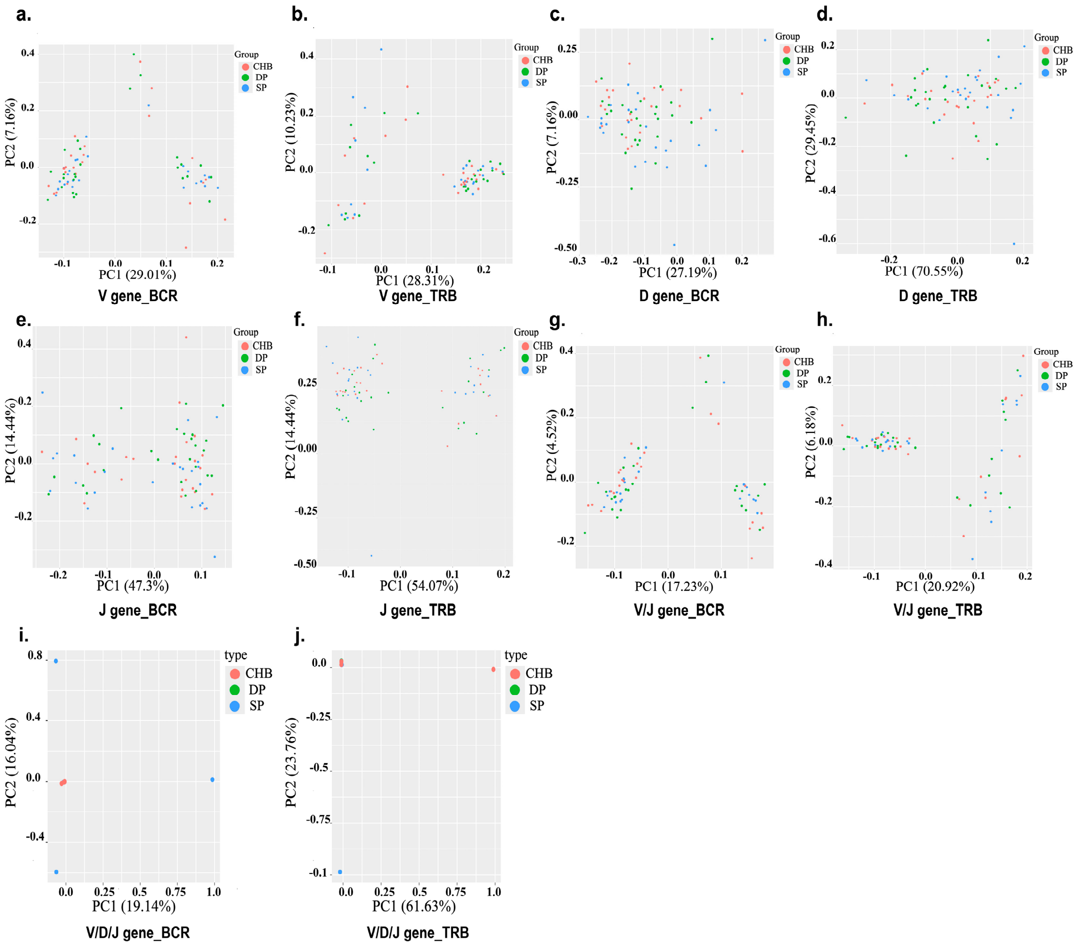

Supplementary Figure S1. Principal component analysis. Principal component analysis (PCA) for BCR and TRB usage of V gene (a, b), D gene (c, d), J gene (e, f), V-D combination (j, h), and V-D-J combination (i, j) in patients with DP group (colored green), SP group (colored blue), and CHB group (colored red) was illustrated. Abbreviations: BCR, B-cell receptor; TRB, T cell receptor β chain; DP: double positive; SP: single positive; CHB: chronic hepatitis B.

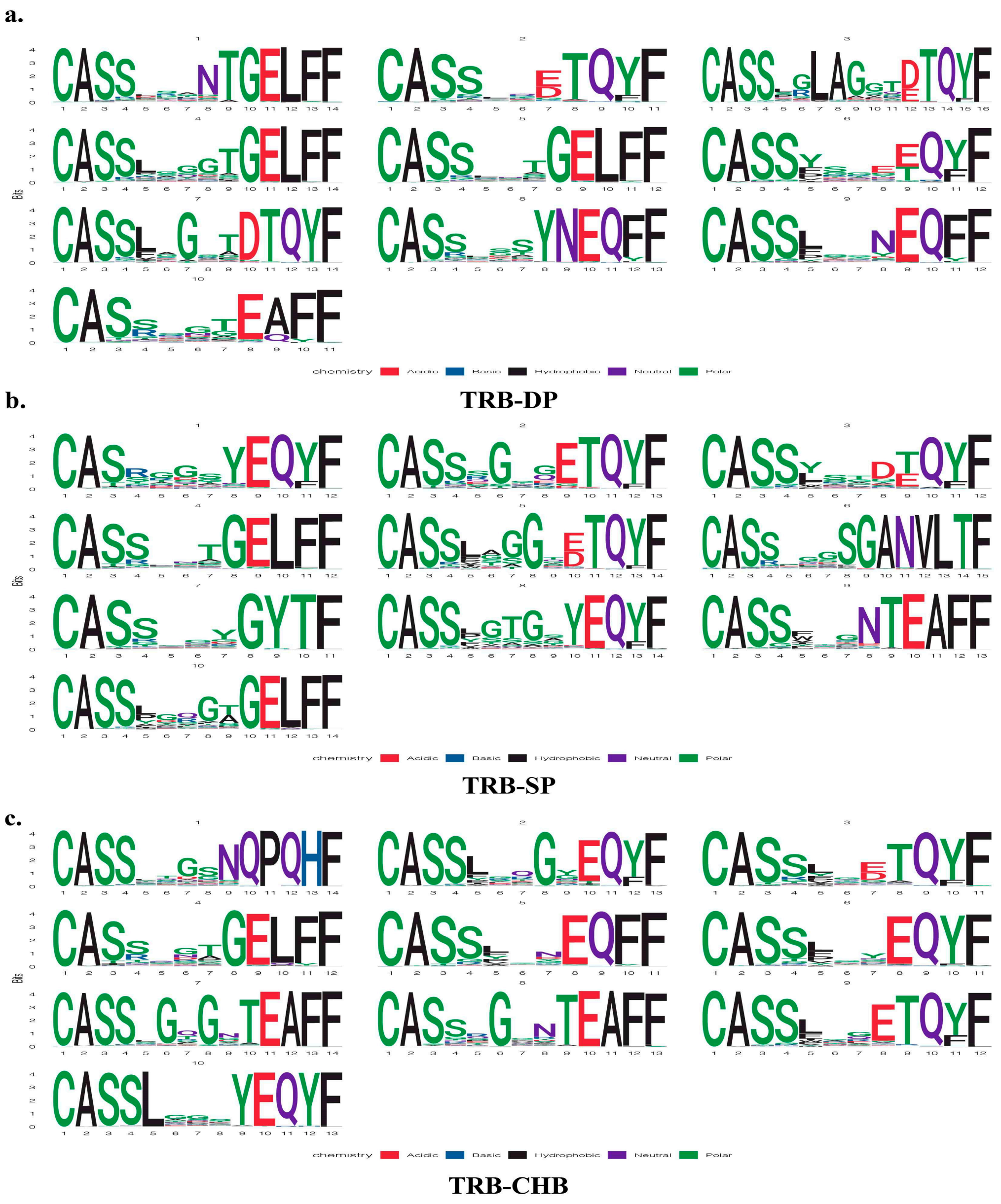

Supplementary Figure S2. Cluster analysis. Sequence logos of annotated top 10 clusters βTCR clonotypes analyzed by clusTCR in DP (a), SP (b), and CHB (c) groups. The label on x-axis represented the positions of CDR3 amino acid and y-axis represented the bits. Different colors represented different types of amino acid. Abbreviations: TRB, T cell receptor β chain; DP: double positive; SP: single positive; CHB: chronic hepatitis B.
